# Supplementary material for: Mechanisms Underpinning Morphogenesis of a Symbiotic Organ Specialized for Hosting an Indispensable Microbial Symbiont in Stinkbugs
Source: mBio. 2023 Apr 5;14(2):e00522-23. doi: 10.1128/mbio.00522-23 (PMC10127593; doi:10.1128/mbio.00522-23)
Supplement: FIG S1 [file mbio.00522-23-s0002.pdf]

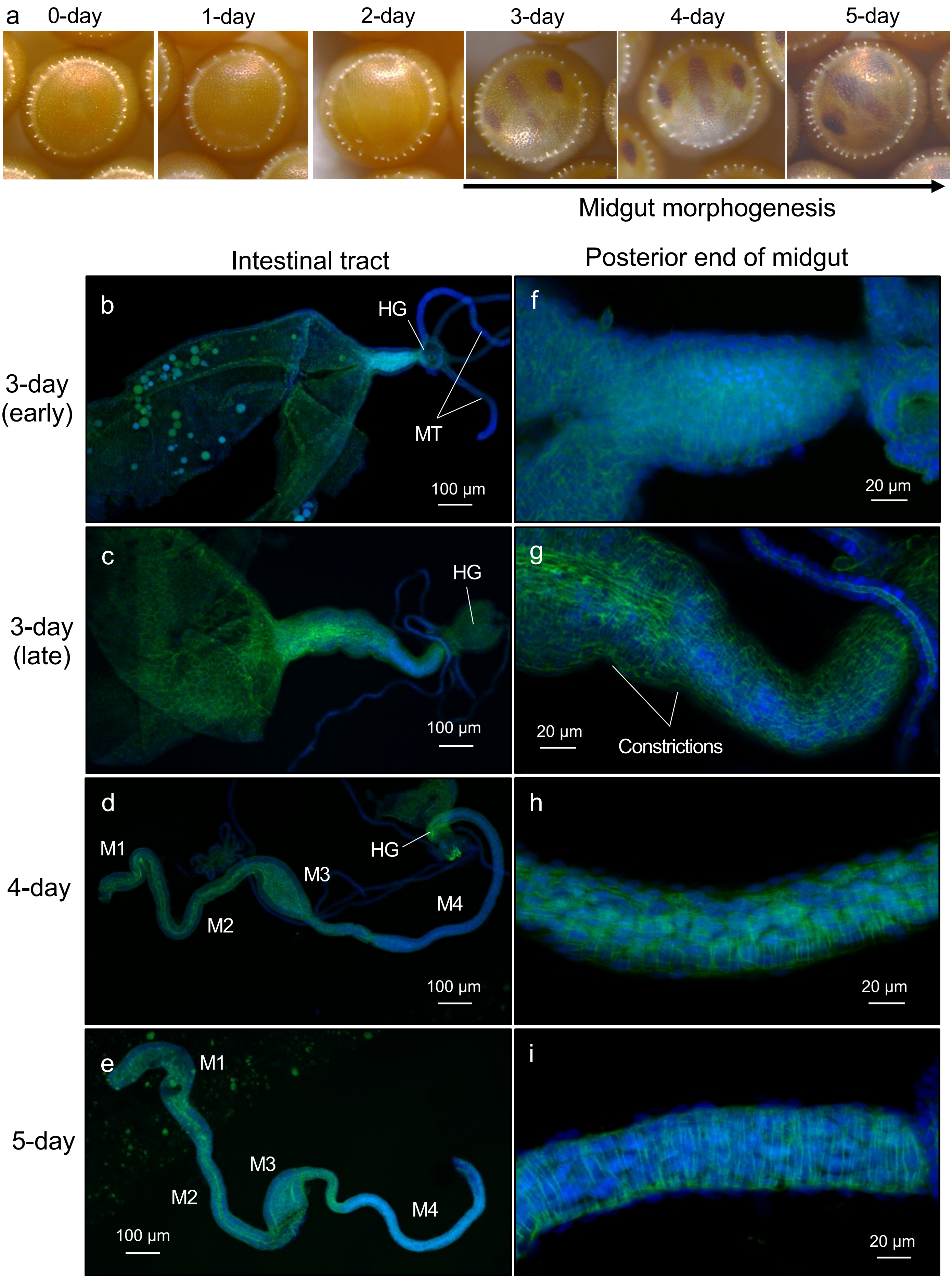

a
0-day
1-day
2-day
3-day
4-day
5-day
Midgut morphogenesis
Intestinal tract
Posterior end of midgut
3-day (early)
3-day (late)
4-day
5-day
b
HG
MT
100 µm
f
20 µm
c
HG
100 µm
g
20 µm
Constrictions
d
M1
M2
M3
M4
HG
100 µm
h
20 µm
e
M1
M2
M3
M4
100 µm
i
20 µm

FIG S1 Embryonic development of the alimentary tract of *P. stali*. (a) Eggs after 0-day, 1-day, 2-day, 3-day, 4-day and 5-day after oviposition. The eggs of *P. stali* usually hatch in five days after oviposition under our rearing condition (19). From the upper side of the eggs, eyes and an egg tooth become visible from 3-day after oviposition and on, during which the development of the alimentary tract proceeds. (b-e) Embryonic alimentary tracts dissected from the developing eggs. (f-i) Enlarged images of the posterior end region of the alimentary tract. (b, f) In early 3-day embryos, the hindgut and the Malphigian tubules are formed, whereas the midgut is still rudimentary. Muscle fibers are still unrecognizable. (c, g) In late 3-day embryos, the midgut exhibits constrictions, presumably reflecting differentiation of midgut regions. Some muscle fibers start to form. (d, h) In 4-day embryos, the midgut M1, M2, M3 and M4 regions are formed, with muscle fibers evidently seen. In the M4 region, circular muscles become evident. (e, i) In 5-day embryos prior to hatching, morphogenesis of the alimentary tract almost completes, with circular and longitudinal muscle fibers well-developed in the M1, M2 and M3 regions. In the M4 region, the bifurcating patterns of circular muscles, as observed in newborn nymphs (see **Fig. 3**), are seen. In (b-i), actin fibers (green) and cell nuclei (blue) are visualized by phalloidin staining and DAPI staining, respectively.
