# Supplementary material for: Mechanisms Underpinning Morphogenesis of a Symbiotic Organ Specialized for Hosting an Indispensable Microbial Symbiont in Stinkbugs
Source: mBio. 2023 Apr 5;14(2):e00522-23. doi: 10.1128/mbio.00522-23 (PMC10127593; doi:10.1128/mbio.00522-23)
Supplement: FIG S2 [file mbio.00522-23-s0001.pdf]

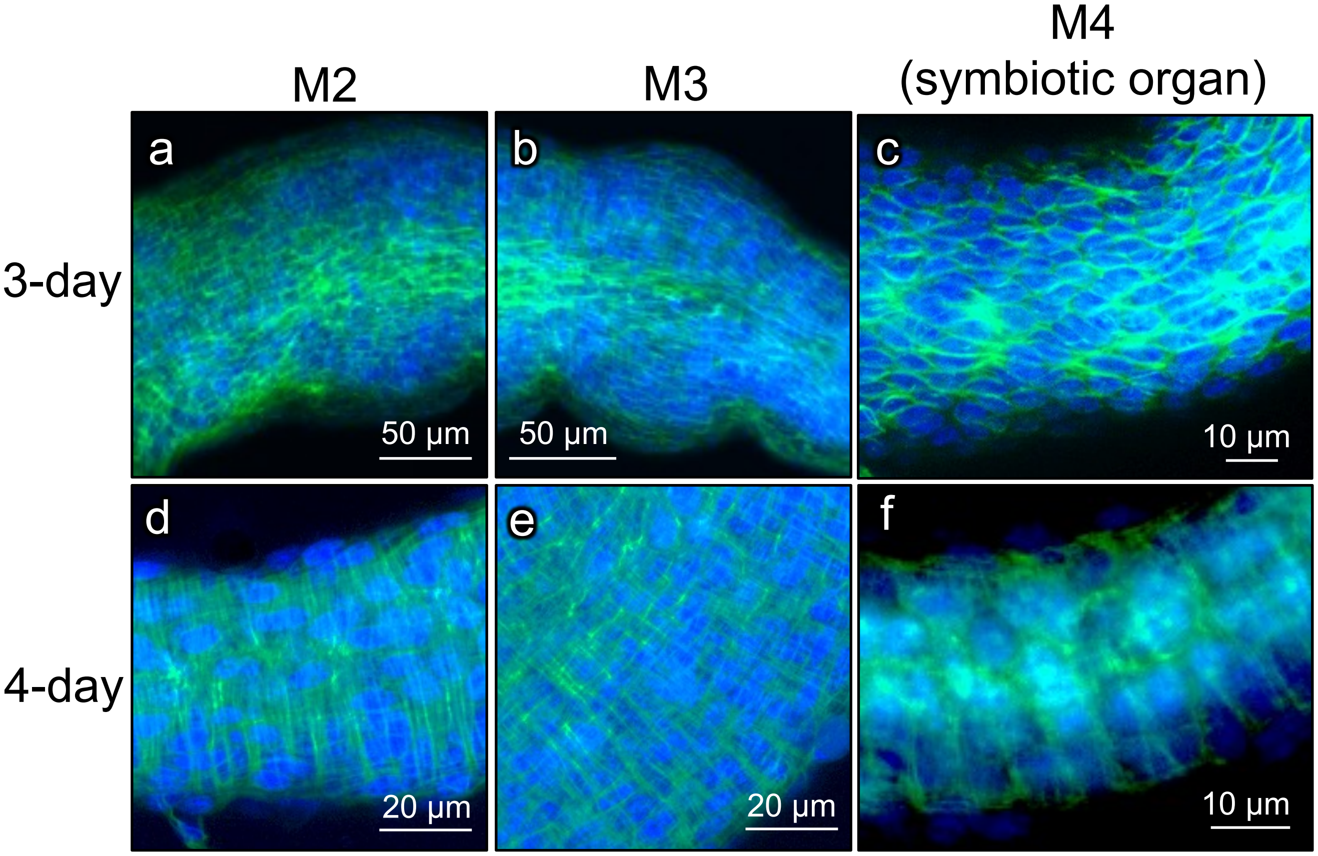

FIG S2 Development of visceral muscle fibers during embryogenesis of *P. stali*. (a-c) Actin fibers (green) and nuclear DNA (blue) visualized in M2 (a), M3 (b) and symbiotic M4 (c) regions of 3-day embryos. Visceral muscles are obscure. (d-f) Actin fibers (green) and nuclear DNA (blue) visualized in M2 (d), M3 (e) and symbiotic M4 (f) regions of 4-day embryos. Circular and longitudinal muscle fibers are clearly seen.

Fig. S2
